# Estimation of the Disease Burden Attributable to 11 Risk Factors in Hubei Province, China: A Comparative Risk Assessment

**DOI:** 10.3390/ijerph13100944

**Published:** 2016-09-23

**Authors:** Fangfang Cui, Lan Zhang, Chuanhua Yu, Songbo Hu, Yunquan Zhang

**Affiliations:** 1 Department of Epidemiology and Biostatistics, School of Health Sciences, Wuhan University, #185 Donghu Road, Wuhan 430071, China; cuifangfang@whu.edu.cn (F.C.); husbo0910@sohu.com (S.H.); Yun-quanZhang@whu.edu.cn (Y.Z.); 2 Office of Chronic Disease, Hubei Province Center for Disease Control and Prevention, #6 Zhuodaoquan Road, Wuhan 430079, China; hbcdczl@163.com; 3 Global Health Institute, Wuhan University, #185 Donghu Road, Wuhan 430071, China

**Keywords:** risk factors, burden of disease, death, disability-adjusted life years

## Abstract

In order to estimate the health losses caused by common risk factors in the Hubei province, China, we calculated the deaths and disability-adjusted life years (DALYs) attributable to 11 risk factors. We estimated the exposure distributions of risk factors in Hubei Province in 2013 from the monitoring system on chronic disease and related risk factors, combined with relative risk (RR) in order to calculate the population attributable fraction. Deaths and DALYs attributed to the selected risk factors were then estimated together with cause-specific deaths and DALYs. In total, 53.39% of the total deaths and 36.23% of the total DALYs in Hubei were a result of the 11 selected risk factors. The top five risk factors were high blood pressure, smoking, high body mass index, diet low in fruits and alcohol use, accounting for 14.68%, 12.57%, 6.03%, 3.90% and 3.19% of total deaths, respectively, and 9.41%, 7.22%, 4.42%, 2.51% and 2.44% of total DALYs, respectively. These risk factors, especially high blood pressure, smoking and high body mass index, significantly influenced quality of life, causing a large number of deaths and DALYs. The burden of chronic disease could be substantially reduced if these risk factors were effectively controlled, which would allow people to enjoy healthier lives.

## 1. Introduction

With the decline in mortality from communicable diseases, chronic non-communicable diseases (NCDs) have replaced communicable diseases as the main risks to population health [1,2]. Detailed descriptions of the mortality, prevalence and causes of NCDs are important for improving population health. Risk factors, such as smoking, drinking and physical inactivity have a well-established relationship with NCDs [3,4,5]. Measuring the disease burden attributable to risk factors can help identify the main drivers of health and can inform on prevention efforts by providing an overview of the corresponding health losses.

Over the past few decades, both the life expectancy and the healthy life expectancy of the Chinese population have increased rapidly, to 76.5 years and 67.9 years in 2013, respectively [6]. However, the gap between healthy life expectancy and life expectancy remains large, and the main reason for this gap is the prevalence of NCDs. The current leading causes of reduced life expectancy are cardiovascular diseases, malignancies, respiratory diseases and injuries [7,8]. All of these diseases are closely related to unhealthy lifestyle and poor dietary habits; therefore, in order to improve life expectancy, it is essential to control these risk factors. Calculating the disease burden caused by risk factors provides evidence on how many deaths and disability-adjusted life years (DALYs) could be averted by controlling these risk factors, which can help inform people and encourage them to change unhealthy lifestyle behaviors.

Previous studies on risk factors have mostly aimed to assess the effects of these factors on the incidence and mortality of disease [9,10,11], whereas few studies have addressed the effects on disease burden. Murray and Lopez provided an important contribution to the study of the disease burden due to risk factors by evaluating the disease burden of ten major risk factors in 1990 for the first time globally [12]. Subsequently, they proposed a conceptual framework for the comparative quantification of health risks [13]. They are currently conducting the global burden of disease study (GBD) [14,15]. The GBD 2013 provided a comparative assessment of 76 risk factors or clusters of risks for 188 countries from 1990 to 2010, including in China [16]. However, it only evaluated the disease burden of risk factors at the national level, and did not perform assessments at the subnational level. China has 34 provinces, and because of the differences in geography, economy and social customs, the population distribution of risk factors exposure varies substantially in different provinces. Therefore, the disease burden caused by risk factors in China overall cannot represent the conditions in Hubei Province.

In addition to the GBD study, other researchers have examined the disease burden of risk factors. Eric et al. assessed morbidity and mortality attributable to alcohol, tobacco and illicit drug use in Canada [11]. Schneider et al. estimated the disease burden attributable to alcohol use in South Africa in 2000 [17]. Mandy et al. analyzed the burden of stroke attributable to selected lifestyle risk factors in rural South Africa [18]. These studies provided an evaluation of a few risk factors or calculated the deaths caused by risk factors. However, they did not assess DALYs for risk factors. In China, there are a few studies on the disease burden caused by risk factors. Xu et al. made an estimation of the mortality and disease burden attributed to 19 risk factors in Shandong Province based on the data from the GBD 2001 [19]. Deng et al. calculated the loss of life expectancy caused by risk factors in Ningxia, China [20]. Chen et al. analyzed DALY and economic burden due to smoking in Hangzhou city in 2013 [21]. Studies on the disease burden attributable to risk factors in Hubei have not been seen.

Risk factors are the primary causes of many diseases, especially NCDs, and therefore reliable and comparable analyses of their risks to health are key to preventing disease and injury. Very few studies have been conducted on the disease burden attributable to risk factors in the provinces or districts of China, including Hubei Province. However, sufficient data are now available to quantify the burden of disease due to risk factors in Hubei Province. In this article, we describe the findings of the disease burden attributable to 11 risk factors in Hubei Province in 2013.

## 2. Materials and Methods

### 2.1. Data Sources

Calculating the disease burden attributable to risk factors requires four types of data: demographic data, mortality data, DALYs caused by disease and injury, and data on the distribution of exposure to risk factors in the population. The demographic data were collected from surveillance of the population conducted by the Hubei Province center for disease control and prevention. Mortality data were acquired from a death surveillance system in Hubei Province in 2013 and were classified according to the ICD-10. We multiplied the DALY rate for Hubei Province by the population to obtain DALYs caused by diseases and injuries in 2013. The DALY rates cited in this paper were from GBD 2013 (http://vizhub.healthdata.org/gbd-compare/), which provided credible estimates of DALYs for 188 countries and subnational regions of China [6], and are shown in Table A1.

Data regarding the rate of exposure to the 11 risk factors were obtained from the monitoring system on chronic diseases and related risk factors in Hubei Province in 2013. The monitoring subjects were collected from six countries in Hubei Province using stratified cluster sampling method, and a total of 3600 inhabitants completed the questionnaire. In addition, we selected the diseases and injuries related to each risk factor based on meta-analyses [22,23], epidemiological studies [24], and the GBD study [16], and we also obtained the relative risk (RR) between diseases and risk factors from these studies. For different exposure levels and sexes, the values of RR differed. A list of diseases or injuries caused by risk factors is shown in Table 1.

### 2.2. Methods

The contribution of a risk factor to disease burden was estimated by comparing the burden due to the observed exposure distribution in a population with the burden that would arise from a hypothetical exposure distribution. The method we used in this paper was thus counterfactual analysis, which has been described in detail elsewhere [13,14]. First, we combined RR with the rate of exposure to risk factors to derive the population attributable fraction (PAF). The PAF represents the fraction of disease burden from a cause that is attributed to the exposure to a risk factor. For continuous risk factors, we used the following formula to calculate PAF.

(1) $$PAF=\frac{\overset{n}{\underset{i=0}{\int }}P_{i}RR_{i}d_{i}-\overset{n}{\underset{i=0}{\int }}{P^{\prime }}_{i}RR_{i}d_{i}}{\overset{n}{\underset{i=0}{\int }}P_{i}RR_{i}d_{i}}$$

where $P_{i}$ and ${P^{\prime }}_{i}$ are the current and counterfactual distributions of risk factor exposure, respectively, $RR_{i}$ is the relative risk of a given cause at exposure level *i*, *i* is the different level of exposure and *n* is the top exposure level. To estimate PAF for categorical risk factors, we used a different formula.

(2) $$PAF=\frac{\sum_{j=1}^{m}P_{j}(RR_{j}-1)}{\sum_{j=1}^{m}P_{j}(RR_{j}-1)+1}$$

where $RR_{j}$ is the RR for exposure category *j*, $P_{j}$ is the exposure rate in the population for exposure category *j*, and *m* is the total number of exposure categories.

Then, we multiplied the PAF by DALYs or deaths to estimate the attributable DALYs and deaths for a cause due to exposure to a given risk factor. The equation used to calculate the DALYs attributable to a risk factor was as follows.

(3) $$AB_{x}=\sum_{d}B_{d}*PAF_{xd}$$

where $AB_{x}$ is the DALYs attributable to risk factor *x*, $B_{d}$ is DALYs of cause *d*, and $PAF_{xd}$ is the fraction of disease burden for cause *d* due to risk factor *x*. When we input the deaths of cause *d* as $B_{d}$, $AB_{x}$ represents the deaths caused by risk factor *x*.

The disease burden attributable to a risk factor was equal to the sum of the attributable burden for each cause related to that risk factor.

## 3. Results

The deaths attributable to the 11 risk factors in Hubei Province in 2013 are listed in Table 2. The total number of deaths caused by the 11 risk factors in both sexes was 191,335, accounting for 53.39% of all deaths, with 129,938 deaths in males, representing 61.61% of all male deaths, and 61,397 in females, corresponding to 41.16% of all female deaths. The mortality rates for males, females and both sexes were 437.21, 217.18 and 329.94 per 100,000 people, respectively. The leading risk factor for death was high blood pressure, accounting for 52,778 deaths and 14.68% of total deaths, followed by smoking (45,186 deaths and 12.57% of total deaths), high body mass index (21,668 deaths and 6.03% of total deaths), diet low in fruits (14,026 deaths and 3.90% of total deaths), and alcohol use (11,462 deaths and 3.19% of total deaths). Of the main risk factors, the top five caused 40.37% of total deaths. The order of the leading risk factors of death differed by sex. The top five risk factors for death in males were, in order, smoking, high blood pressure, high body mass index, alcohol use and diet low in fruit, whereas for females, instead of alcohol use, diet high in sodium was one of the first five risk factors. Furthermore, smoking and alcohol use were ranked much higher in males than in females.

**Figure 1:**
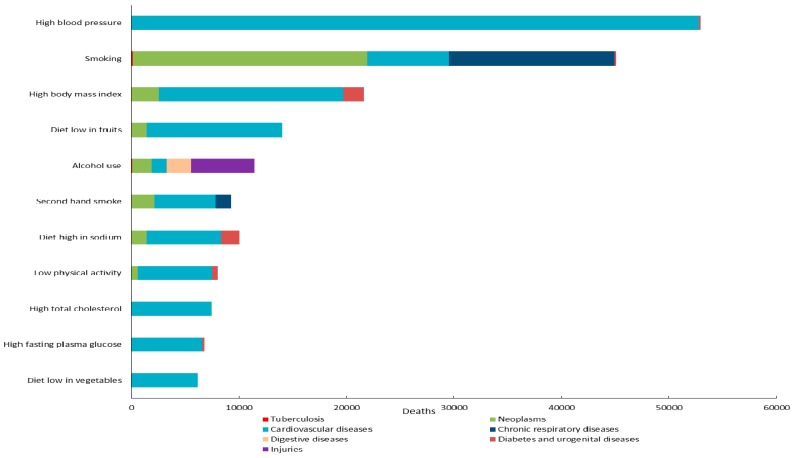
Main causes of deaths attributed to each risk factor for both sexes in Hubei Province in 2013.

Figure 1 shows the main causes of death related to each risk factor. All of the risk factors contributed to the deaths of cardiovascular diseases, especially high blood pressure, high body mass index and diet low in fruits. As the leading risk factor, 99.63% of deaths caused by high blood pressure were related to cardiovascular diseases. High body mass index, diet high in sodium, and low physical activity were also related to diabetes and urogenital diseases, with 9.09%, 16.92% and 6.93% of deaths caused by the disease, respectively. In addition, smoking was a leading risk factor for neoplasms and chronic respiratory diseases, with 48.44% and 34.08% of deaths related to the two diseases, respectively. In contrast to smoking, most deaths attributable to second-hand smoke were associated with cardiovascular diseases. Injuries were related to 51.21% of deaths caused by alcohol use. Although the number of deaths attributable to other risk factors were small when compared to those for high blood pressure and smoking, they were associated with much greater disease burden from NCDs. All deaths caused by high total cholesterol and 96.86% of deaths from high fasting plasma glucose were associated with cardiovascular diseases.

Table 3 depicts the DALYs caused by each risk factor. The DALY rate attributable to the 11 risk factors for both sexes was 9624.04 per 100,000 people, and it was 13,443.22 per 100,000 people for males, which was much higher than the 5609.00 per 100,000 people rate in females. All the risk factors included in this paper accounted for 36.48% of the total DALYs overall, 45.20% of the total DALYs for men, and 24.20% of the total DALYs for women. As with the top three risk factors for deaths, the first three risk factors for DALYs remained high blood pressure (9.41% of total DALYs), smoking (7.22% of total DALYs), and high body mass index (4.67% of total DALYs). However, the last risk factor was diet low in vegetables, accounting for 1.07% of the total DALYs. The order of risk factors for DALYs varied by population. The top three risk factors for males were smoking (accounting for 11.58% of the total DALYs), high blood pressure (10.97% of total DALYs), and alcohol use (4.15% of total DALYs). For women, the first three risk factors were high blood pressure (representing 7.33% of total DALYs), high body mass index (5.15% of total DALYs) and diet low in fruits (1.97% of total DALYs), whereas smoking (1.35% of total DALYs) and alcohol use (0.13% of total DALYs) ranked 8th and last, respectively.

**Figure 2:**
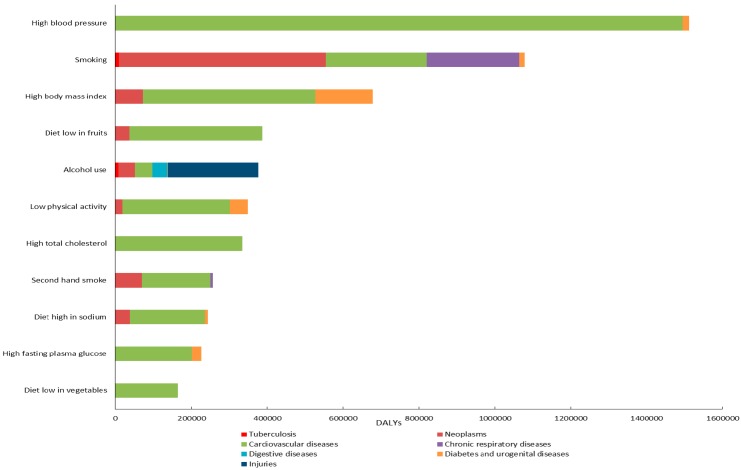
Main causes of DALYs attributed to each risk factor for both sexes in Hubei Province in 2013.

From Figure 2, we can identify the causes of DALYs attributed to each risk factor. Regarding metabolic risk factors, the DALYs caused by high blood pressure, high body mass index, high total cholesterol and high fasting plasma glucose primarily originated from cardiovascular diseases and urogenital diseases. The single largest risk factor for DALYs of cardiovascular diseases was high blood pressure, responsible for an estimated 149,000 cardiovascular DALYs. High body mass index contributed most to the disease burden of urogenital diseases, accounting for an estimated 151,000 urogenital DALYs. The behavioral risk factors in this paper, such as smoking, second hand smoke, and diet low in fruits or vegetables, mainly caused disease burden due to neoplasms and cardiovascular diseases. Compared with any other risk factor, smoking had the largest effect on the disease burden of neoplasms, causing an estimated 545,000 neoplasm-related DALYs. The majority of DALYs attributable to low physical activity and diet high in sodium were related to cardiovascular diseases. As the single risk factor related to digestive diseases in this paper, alcohol use caused an estimated 40,000 digestive DALYs and 236,000 DALYs of injuries.

## 4. Discussion

As one of the first studies to quantitatively estimate the disease burden attributable to risk factors in one province of China, we used deaths and DALYs as metrics to evaluate the burden of disease caused by 11 common risk factors. We found that the 11 risk factors accounted for about half of the total deaths and a third of the total DALYs in 2013, indicating that these factors are highly harmful to population health; these findings help inform the government of the need to take action to control these risk factors. As the top two risk factors, high blood pressure and smoking contributed substantially to population deaths and DALYs, which is consistent with the findings worldwide and in the U.S. [25,26,27]. The ranking of the 11 risk factors based on deaths was high blood pressure, followed by smoking, high body mass index, diet low in fruits, alcohol use, second hand smoke, diet high in sodium, low physical activity, high total cholesterol, high fasting plasma glucose and diet low in vegetables, which is similar to the order of risk factors in the Asian-Pacific region reported in the GBD study of 2013 [16]. However, second hand smoke ranked higher in Hubei Province, and thus more attention should be paid to decrease the prevalence of second hand smoke in Hubei Province.

Smoking accounted for 19.71% of deaths and 11.58% of DALYs in males, while the corresponding proportions were only 2.43% and 1.35% in females, respectively. Similarly, alcohol use caused a much greater burden of disease in males than in females. These findings indicate that males are a high-risk population for smoking and alcohol use in Hubei Province, which is in line with the results of most studies in China [28,29]. In contrast with males, high body mass index and diet high in sodium ranked higher in females, and thus specific health promotion and education efforts should be implemented for different sexes. Additionally, the disease burden among males was greater than that of females; this is probably because women have a greater interest and pay greater attention to health issues [30]. This finding also reminds us that greater attention should be paid to the prevention and control of risk factors among males.

Moreover, we found that the main diseases related to the studied risk factors were chronic diseases, especially cardiovascular diseases and neoplasms, which were the leading causes of reduced life expectancy. Therefore, preventing the occurrence of risk factors can decrease the morbidity of chronic diseases and improve life expectancy. Second hand smoke caused a large number of deaths and is strongly related to the high prevalence of smoking; thus, emphasis should be placed on creating and maintaining smoke-free zones, as well as on smoking prevention and control. Smoking and second hand smoke were the main risk factors for chronic respiratory diseases, such as COPD; in the absence of smokers, the deaths due to chronic respiratory disease could be decreased by 15.16%. Aiming to decrease the disease burden attributable to smoking, the Hubei Province government issued a circular in 2014, ordering officials at all levels to stop smoking in offices and public places; however, the government has not banned smoking for whole population in public places until now, and the prevalence of smoking is still high in Hubei Province. Unlike Hubei, Beijing Municipal Government enacted a stricter regulation against smoking in public places and indoor workplaces for all people in Beijing, and fined those who did not comply. After the implementation of the antismoking policies for one year, the number of smokers in public places decreased significantly, and population’s awareness of tobacco control was raised. China has the world’s largest smoking population [31], and although the tobacco tax increased in 2009 and 2015, it was insufficient to induce smokers to give up purchasing tobacco products. Thus, only total bans in public places can reduce the number of smokers.

As the leading risk factor, more than ninety percent of disease burden attributable to high blood pressure was related with cardiovascular diseases. A decrease in population blood pressure will greatly reduce the mortality of cardiovascular diseases, such as stroke, the top cause of death in China [32]. Community knowledge, treatment rate and control rate of hypertension are still low in China, and the potential key factors for the decline of blood pressure may include increasing use of blood pressure medication and changing unhealthy life styles [33]. Morbidity relating to hypertension in youth has increased in recent years therefore, it suggested that all the hypertension patients should be involved in regular management, and health education for younger patients needs to be strengthened.

According to previous studies [34,35,36], alcohol use is one of the leading risk factors for injuries, road traffic injuries in particular. In our study, we found that almost half of the disease burden attributable to alcohol use was related to injuries. Effective drunk driving laws and banning some alcohol advertising can be useful for reducing injuries caused by alcohol use. Our study also suggests that low physical activity contributes to substantial mortality due to cardiovascular disease and diabetes in Hubei Province. Risk factor surveillance has adopted the global physical activity questionnaire (GPAQ) for collecting information about activity at work, transportation and recreational activity, showing that the exposure rate is highest for those who lack recreational activity. Considering that physical activity is related to many NCDs, it’s important to improve people’s physical consciousness on exercise and provide support for exercise in public places, such as in schools and in the community. In order to control the other risk factors, an effective method is to publicize healthy practices and improve public understanding of the role of risk factors in disease prevention. For different risk factors, the main related diseases varied; for example, people with a high body mass index, high fasting plasma glucose and high total cholesterol were vulnerable to cardiovascular diseases, while those who ate few fruits had high risks of neoplasms. Therefore, we shall put more emphasis on the high risk population when taking action on disease control, which may have a better effect.

The study has some limitations. Firstly, as the risk factor surveillance system was still in its initial development stage, we could only collect exposure data for 11 risk factors, and thus the ranking of risk factors was relative and not representative of the ranking of all risk factors. Secondly, because of the difficulty in collecting data, especially prevalence data of diseases, we did not calculate the DALYs for each disease; rather, we used the DALY results of the GBD 2013. In the future, we will aim to collect more data in order to estimate the DALYs for diseases ourselves, as well as to evaluate the disease burden for more risk factors. Thirdly, we assumed that the risk factors were independent of each other and did not take into consideration the interactions between the risk factors; this may have led to a slightly higher disease burden attributable to each risk factor. Therefore, the interactions between risk factors are the focus of our future research.

## 5. Conclusions

The results of this paper suggest that the 11 risk factors studied accounted for 53.39% of total deaths and 36.23% of total DALYs, and therefore present a great risk to population health in the Hubei Province; high blood pressure and smoking in particular caused a greater number of deaths and DALYs than the other risk factors. There was a higher total number of deaths and DALYs attributable to the 11 risk factors in males than in females. The main risk factors for males were smoking, high blood pressure, alcohol use and high body mass index, however, the major risk factors were high blood pressure, high body mass index, diet low in fruits and low physical activity in females. All the risk factors in this paper could cause cardiovascular diseases, especially high blood pressure, high total cholesterol and high fasting plasma glucose. The behavioral risk factors, such as smoking and diet low in fruits, had a greater relationship to neoplasms; alcohol use was a main risk factor for injuries. Controlling these risk factors could make great contribution towards reducing the prevalence of NCDs.

## Figures and Tables

**Table 1:** Main diseases and injuries related to each risk factor.

Risk Factor	Disease and Injury Outcomes
Smoking	Cancer of the upper digestive tract, liver cancer, stomach cancer, lung cancer, cervical cancer, colorectal cancer, pancreatic cancer, kidney cancer, bladder cancer, leukemia, COPD, pneumoconiosis, interstitial lung disease, tuberculosis, lower respiratory infections, other chronic respiratory diseases, ischemic heart disease, ischemic stroke, hemorrhagic stroke, hypertensive heart disease, atrial fibrillation, aortic aneurysm, peripheral vascular disease, other cardiovascular and circulatory diseases, asthma, diabetes
Second hand smoke	Lung cancer, lower respiratory infections, otitis media, ischemic heart disease, ischemic stroke, hemorrhagic stroke
Alcohol use	Cancer of the upper digestive tract, liver cancer, breast cancer, colorectal cancer, alcoholic cirrhosis, pancreatitis, tuberculosis, diabetes, ischemic heart disease, ischemic stroke, hemorrhagic stroke, other cardiovascular and circulatory diseases, road injuries, unintentional injuries, self-harm
Diet low in vegetables	Ischemic heart disease, ischemic stroke, hemorrhagic stroke
Diet low in fruits	Cancer of the upper digestive tract, lung cancer, ischemic heart disease, ischemic stroke, hemorrhagic stroke
Diet high in sodium	Stomach cancer, rheumatic heart disease, ischemic heart disease, ischemic stroke, hemorrhagic stroke, hypertensive heart disease, cardiomyopathy, atrial fibrillation, aortic aneurysm, peripheral vascular disease, endocarditis, other cardiovascular and circulatory diseases, diabetes-related CKD, hypertensive CKD, glomerulonephritis-related CKD, other CKD
Low physical activity	Breast cancer, colorectal cancer, ischemic heart disease, ischemic stroke, diabetes
High body mass index	Esophageal cancer, liver cancer, breast cancer, uterine cancer, ovarian cancer, colorectal cancer, gallbladder cancer, pancreatic cancer, kidney cancer, thyroid cancer, leukemia, ischemic heart disease, ischemic stroke, hemorrhagic stroke, hypertensive heart disease, cardiomyopathy, atrial fibrillation, aortic aneurysm, peripheral vascular disease, endocarditis, other cardiovascular and circulatory diseases, diabetes, diabetes-related CKD, hypertensive CKD, glomerulonephritis-related CKD, other CKD, osteoarthritis
High fasting plasma glucose	Ischemic heart disease, ischemic stroke, hemorrhagic stroke, diabetes CKD, hypertensive CKD, glomerulonephritis-related CKD, other CKD
High total cholesterol	Ischemic heart disease, ischemic stroke
High blood pressure	Rheumatic heart disease, hypertensive heart disease, ischemic heart disease, ischemic stroke, hemorrhagic stroke, cardiomyopathy, atrial fibrillation, aortic aneurysm, peripheral vascular disease, endocarditis, other cardiovascular and circulatory diseases, diabetes-related CKD, glomerulonephritis-related CKD, other CKD

COPD = chronic obstructive pulmonary disease; CKD = chronic kidney disease.

**Table 2:** Deaths attributed to 11 risk factors in Hubei Province in 2013.

Risk Factor	Males		Females		Both Sexes	
	Deaths (%) *	Mortality (Per 100,000)	Deaths (%) *	Mortality (Per 100,000)	Deaths (%) *	Mortality (Per 100,000)
Smoking	41,579 (19.71)	139.90	3607 (2.43)	12.76	45,186 (12.57)	77.92
Second hand smoke	6452 (3.06)	21.71	2849 (1.92)	10.08	9301 (2.59)	16.04
Alcohol use	9963 (4.72)	33.52	1499 (1.01)	5.30	11,462 (3.19)	19.77
Diet low in vegetables	3331 (1.58)	11.21	2807 (1.89)	9.93	6138 (1.71)	10.58
Diet low in fruits	8629 (4.09)	29.03	5396 (3.63)	19.09	14,026 (3.90)	24.19
Diet high in sodium	4799 (2.28)	16.15	3665 (2.47)	12.96	8464 (2.35)	14.60
Low physical activity	4560 (2.16)	15.34	3502 (2.36)	12.39	8062 (2.24)	13.90
High body mass index	10,623 (5.04)	35.74	11,045 (7.43)	39.07	21,668 (6.03)	37.37
High fasting plasma glucose	3735 (1.77)	12.57	3055 (1.89)	10.81	6789 (2.06)	11.71
High total cholesterol	4567 (2.17)	15.37	2894 (1.95)	10.24	7461 (2.07)	12.87
High blood pressure	31,700 (15.03)	106.66	21,078 (14.18)	74.56	52,778 (14.68)	91.01
Total	129,938 (61.61)	437.21	61,397 (41.16)	217.18	191,335 (53.39)	329.94

* % means the proportion of deaths attributable to one risk factor out of total deaths.

**Table 3:** Deaths and disability-adjusted life years (DALYs) attributed to 11 risk factors in Hubei Province in 2013.

Risk Factor	Males		Females		Both Sexes	
	DALYs (%) *	DALY Rate (per 100,000)	DALYs (%) *	DALY Rate (per 100,000)	DALYs (%) *	DALY Rate (per 100,000)
Smoking	1,023,480 (11.58)	3443.75	88,748 (1.35)	313.93	1,112,227 (7.22)	1917.96
Second hand smoke	185,507 (2.10)	624.18	73,036 (1.12)	258.35	258,543 (1.68)	445.84
Alcohol use	367,122 (4.15)	1235.27	8341 (0.13)	29.50	375,463 (2.44)	647.46
Diet low in vegetables	98,370 (1.11)	330.99	66,707 (1.02)	235.96	165,077 (1.07)	284.66
Diet low in fruits	256,977 (2.91)	864.66	129,266 (1.97)	457.25	386,243 (2.51)	666.05
Diet high in sodium	150,498 (1.70)	506.39	92,297 (1.41)	326.48	242,795 (1.58)	418.68
Low physical activity	225,961 (2.56)	760.30	122,041 (1.86)	431.70	348,003 (2.26)	600.11
High body mass index	344,742 (3.90)	1159.97	337,163 (5.15)	1192.65	681,905 (4.42)	1175.90
High fasting plasma glucose	131,753 (1.49)	443.32	94,528 (1.44)	334.37	226,281 (1.47)	390.21
High total cholesterol	241,097 (2.73)	811.23	93,260 (1.42)	329.89	334,357 (2.17)	576.58
High blood pressure	969,806 (10.97)	3263.15	480,282 (7.33)	1698.90	1,450,087 (9.41)	2500.58
Total	3,995,313 (45.20)	13,443.22	1,585,669 (24.20)	5609.00	5,580,981 (36.23)	9624.04

* % means the proportion of DALYs attributable to one risk factor out of total DALYs.

## Appendix A

**Table A1:** DALY rate (per 100,000) of diseases and injuries in Hubei Province in 2013.

Diseases and Injuries	DALY Rate for Males	DALY Rate for Females	Diseases and Injuries	DALY Rate for Males	DALY Rate for Females
Esophageal cancer	380.42	81.86	Lower respiratory infections	291.17	206.84
Stomach cancer	662.8	298.66	Other chronic respiratory diseases	57.55	62.76
Liver cancer	1298.37	387.02	Alcoholic cirrhosis	114.34	21.97
Lung cancer	1390.57	463.69	Pancreatitis	27.56	22.63
Gallbladder cancer	26.97	38.44	Ischemic heart disease	1968.47	1209.08
Thyroid cancer	7.94	12.23	Ischemic stroke	749.2	529.25
Colorectal cancer	294.63	227.37	Hemorrhagic stroke	2540.38	1664.04
Lip and oral cavity cancer	31.78	15.38	Rheumatic heart disease	110.85	162.91
Nasopharynx cancer	87.91	34.08	Hypertensive heart disease	463.2	388.84
Larynx cancer	48.02	6.78	Cardiomyopathy	74.12	62.94
Other pharynx cancer	14.97	3.23	Atrial fibrillation	5.65	3.66
Breast cancer	5.01	278.08	Aortic aneurysm	32.38	8.82
Uterine cancer	0.00	65.01	Peripheral vascular disease	1.7	2.41
Cervical cancer	0.00	153.91	Endocarditis	7.01	4.14
Ovarian cancer	0.00	87.09	Other cardiovascular and circulatory diseases	172.73	212.44
Pancreatic cancer	100.3	68.56	Diabetes	704.53	629.13
Kidney cancer	36.84	19.48	Diabetes-related CKD	55.98	63.9
Bladder cancer	66.96	17.64	Hypertensive CKD	101.7	81.94
Leukemia	174.95	121.51	Glomerulonephritis-related CKD	106.27	92.71
Tuberculosis	194.89	77.25	Other CKD	146.65	138.5
Otitis media	20.44	18.67	Road injuries	1428.94	520.92
Asthma	117.31	118.67	Unintentional injuries	1682.51	667.61
COPD	1425.93	935.55	Self-harm	621.06	581.59
Pneumoconiosis	26.14	2.31	Osteoarthritis	144.04	294.39
Interstitial lung disease	103.7	85.7			

COPD = chronic obstructive pulmonary disease; CKD = chronic kidney disease.
